# Comparative effects of dexmedetomidine and propofol on brain and lung damage in experimental acute ischemic stroke

**DOI:** 10.1038/s41598-021-02608-1

**Published:** 2021-11-30

**Authors:** Giselle C. Sousa, Marcos Vinicius Fernandes, Fernanda F. Cruz, Mariana A. Antunes, Carla M. da Silva, Christina Takyia, Denise Battaglini, Cynthia S. Samary, Chiara Robba, Paolo Pelosi, Patricia R. M. Rocco, Pedro L. Silva

**Affiliations:** 1 Laboratory of Pulmonary Investigation, Carlos Chagas Filho Biophysics Institute, Federal University of Rio de Janeiro, Centro de Ciências da Saúde, Avenida Carlos Chagas Filho, 373, Bloco G-014, Ilha Do Fundão, Rio de Janeiro, RJ 21941-902 Brazil; 2 Department of Anesthesiology, Federal University of Rio de Janeiro, Rio de Janeiro, Brazil; 3 Rio de Janeiro Network on Neuroinflammation, Carlos Chagas Filho Foundation for Supporting Research in the State of Rio de Janeiro (FAPERJ), Rio de Janeiro, Brazil; 4 Laboratory of Imunopathology, Institute of Biophysics Carlos Chagas Filho, Federal University of Rio de Janeiro, Rio de Janeiro, RJ Brazil; 5 San Martino Policlinico Hospital, IRCCS for Oncology and Neurosciences, University of Genoa, Genoa, Italy; 6 Department of Surgical Sciences and Integrated Diagnostics (DISC), University of Genoa, Genoa, Italy

**Keywords:** Neuroscience, Physiology

## Abstract

Acute ischemic stroke is associated with pulmonary complications, and often dexmedetomidine and propofol are used to decrease cerebral metabolic rate. However, it is unknown the immunomodulatory actions of dexmedetomidine and propofol on brain and lungs during acute ischemic stroke. The effects of dexmedetomidine and propofol were compared on perilesional brain tissue and lung damage after acute ischemic stroke in rats. Further, the mean amount of both sedatives was directly evaluated on alveolar macrophages and lung endothelial cells primarily extracted 24-h after acute ischemic stroke. In twenty-five Wistar rats, ischemic stroke was induced and after 24-h treated with sodium thiopental (STROKE), dexmedetomidine and propofol. Dexmedetomidine, compared to STROKE, reduced diffuse alveolar damage score [median(interquartile range); 12(7.8–15.3) vs. 19.5(18–24), *p* = 0.007)], bronchoconstriction index [2.28(2.08–2.36) vs*.* 2.64(2.53–2.77), *p* = 0.006], and TNF-α expression (*p* = 0.0003), while propofol increased VCAM-1 expression compared to STROKE (*p* = 0.0004). In perilesional brain tissue**,** dexmedetomidine, compared to STROKE, decreased TNF-α (*p* = 0.010), while propofol increased VCAM-1 compared to STROKE (*p* = 0.024). In alveolar macrophages and endothelial cells, dexmedetomidine decreased IL-6 and IL-1β compared to STROKE (*p* = 0.002, and *p* = 0.040, respectively), and reduced IL-1β compared to propofol (*p* = 0.014). Dexmedetomidine, but not propofol, induced brain and lung protection in experimental acute ischemic stroke.

## Introduction

Dexmedetomidine (α2-adrenergic agonist) and propofol (non-barbiturate sedative) are commonly used in the clinical management of patients with acute ischemic stroke who require admission to a neurocritical care ward^1^. Both decrease cerebral metabolic rate and present anti-inflammatory, antioxidant and anti-apoptotic properties^2,3^. While propofol reduces intracranial pressure (ICP), dexmedetomidine preserves cerebral metabolic rate (CMR)-cerebral blood flow (CBF) coupling^4,5^. Besides, dexmedetomidine inhibits the presynaptic release of glutamate, aspartate, and norepinephrine, inhibits adenylate and guanylate cyclases and can act through mitochondrial imidazoline receptors on astrocytes in penumbra^2^. Dexmedetomidine has also been associated with a dose-dependent protective effect against the loss of brain matter in vivo and improved neurological functional deficit induced by hypoxic-ischemic insult^6^, which may be related to its action through the α7 nicotinic acetylcholine receptor. Propofol may protect pyramidal neurons in the hippocampal region in global brain ischemia by modulating the binding properties of glutamate receptors and the uptake of glutamate^3^. However, propofol may lead to neurotoxicity effects in traumatic brain injury^7^.

Acute ischemic stroke may lead to death of neuronal cells because of the reduction of energetic substrates. Although brain injury is due to nutrient deprivation, the worst of the neurological deficits is likely due to consequent inflammatory processes^8,9^. In addition to local inflammatory responses in the brain, systemic responses can be observed after stroke, which can have important effects on distal organs^9,10^. An important crosstalk between brain and lungs has been previously described^11^ in this context, as focal ischemic stroke seems to be associated with an increase in lung damage and systemic inflammation^9^. Thus, acute ischemic stroke is associated with a high incidence of pulmonary complications, including ventilator-associated pneumonia, acute respiratory distress syndrome, and neurogenic pulmonary edema^12^, which is likely a consequence of the brain-lung crosstalk^9,13^.

To the best of our knowledge, it is unknown whether the aforementioned immunomodulation in central nervous system (CNS) proffered by dexmedetomidine and propofol during acute ischemic stroke is further extended to the lung. We hypothesized that 24 h after acute ischemic stroke, dexmedetomidine and propofol might promote brain and lung protection with direct effects on pulmonary macrophages and endothelial cells. We compared the effects of dexmedetomidine and propofol (1-h infusion) on the perilesional brain tissue and lungs, also, we assessed respiratory variables, hemodynamic, lung histology, gene expression of inflammatory markers, 24-h after acute ischemic stroke in rats. We also investigated if both anesthetics would act directly on alveolar macrophages and lung endothelial cells primarily extracted 24-h after acute ischemic stroke.

## Results

No differences were observed in respiratory variables and hemodynamic between propofol and dexmedetomidine groups overtime (Supplemental Material, Table S1). In addition, no major changes were observed in blood gas analysis (Supplemental Material, Table S2). Compared to SHAM animals, the STROKE group showed higher cumulative DAD and BI (Supplemental Material, Table S3, Fig. S2), increased TNF-α and VCAM-1 expressions in lung and brain tissues (Supplemental Material, Fig. S3), as well as FJC + , CD45 + , CD68 + cells and ApopTag (Supplemental Material, Fig. S4).

### Lung damage

**Figure 1:**
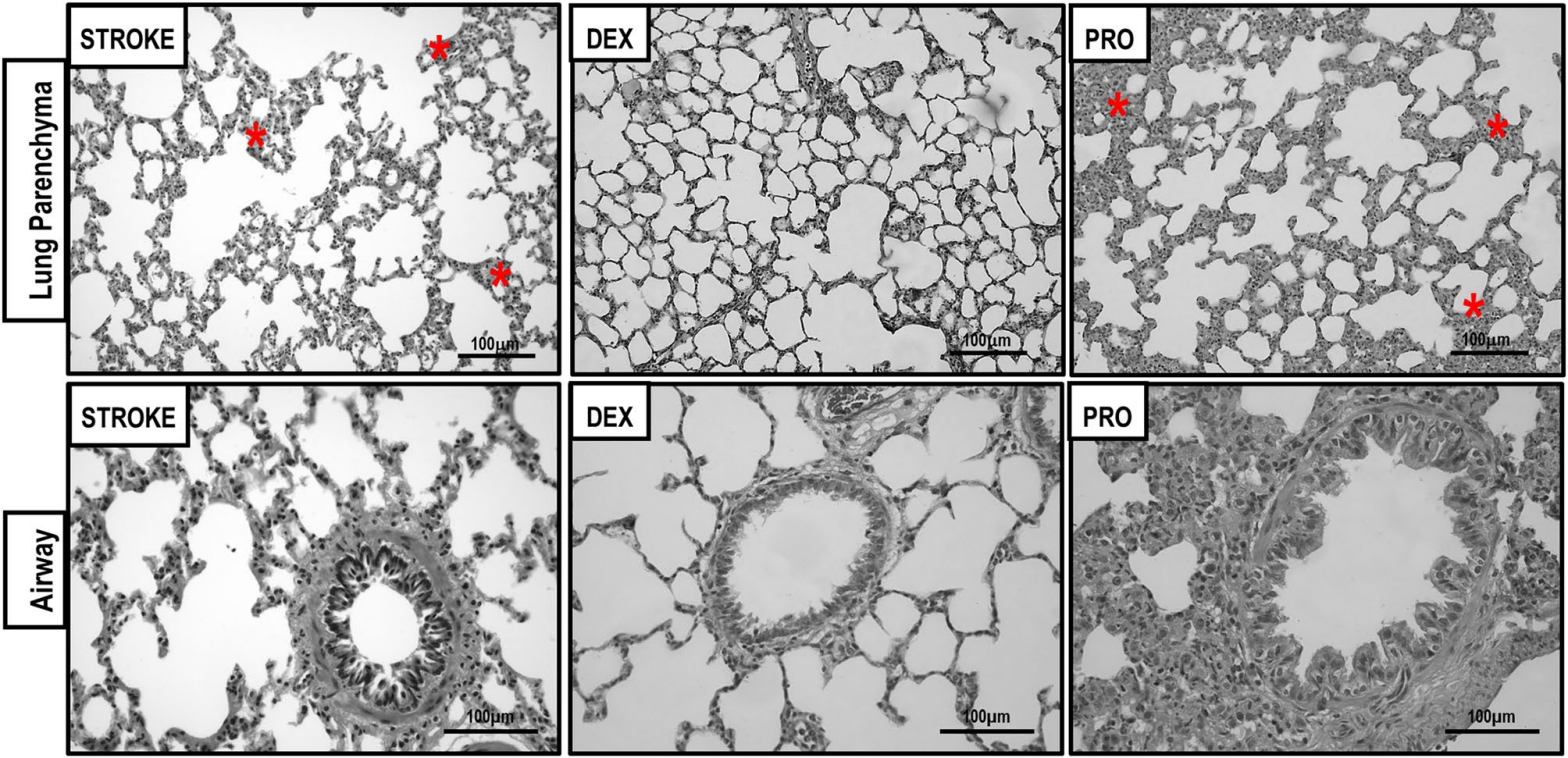
All animals were submitted to focal ischemic stroke. 24-h later, they were randomly assigned, by sealed envelopes, to (1) Group STROKE; (2) Group DEX; and (3) Group PRO. After 1-h of anesthetic infusion, lungs were removed for histology analysis. Representative images of lung parenchyma upon autopsy in STROKE group (only stroke without dexmedetomidine or propofol infusion), after dexmedetomidine and propofol infusion (superior panels, × 200) and airway (inferior panels, × 400). Note the atelectasis (red asterisks) in the STROKE and PRO groups. The airway lumen in STROKE and PRO groups was constricted compared to DEX group. Scale bar = 100 μm.

**Figure 2:**
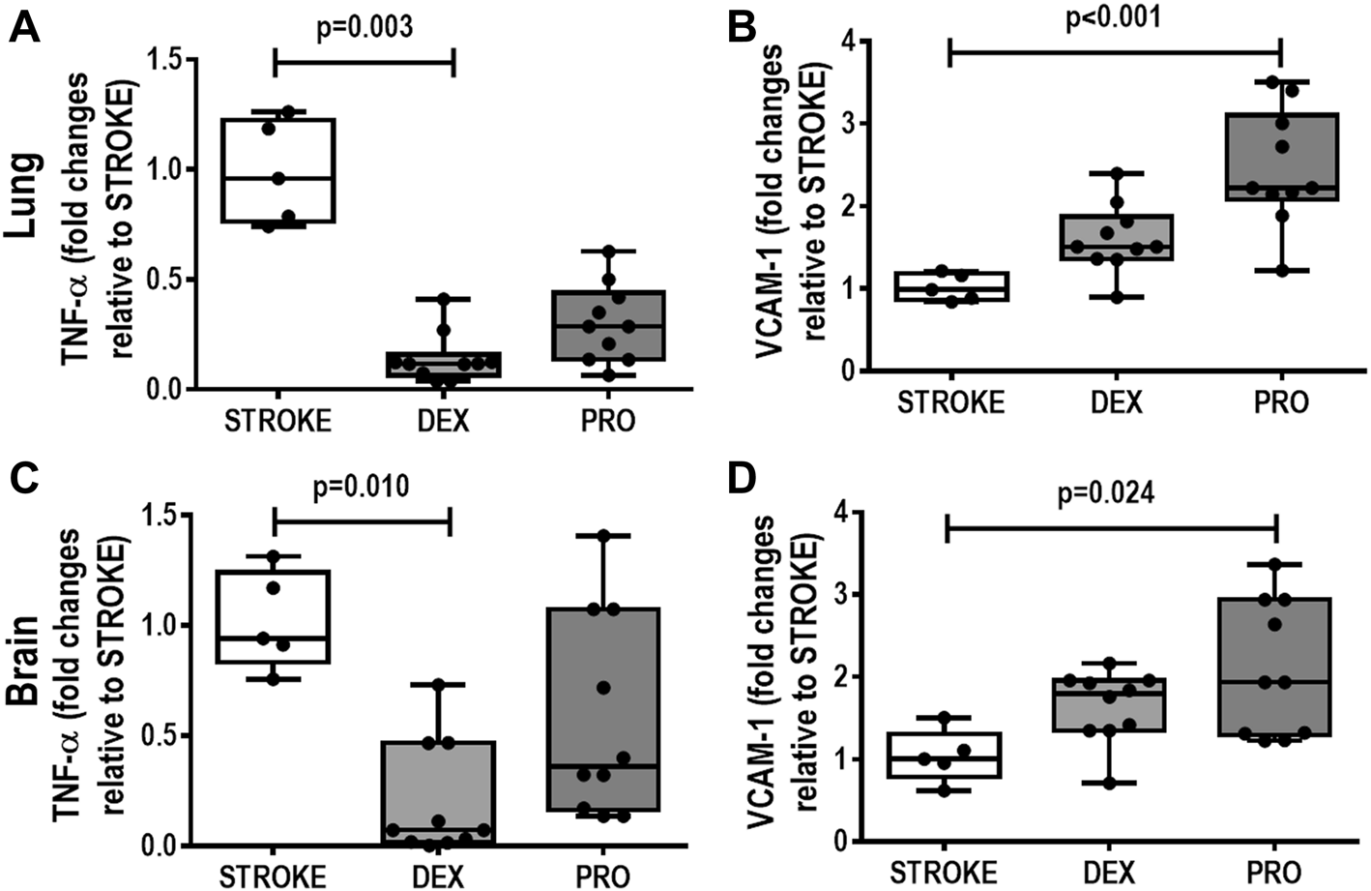
All animals were submitted to focal ischemic stroke. 24-h later, they were randomly assigned, by sealed envelopes, to (1) Group STROKE; (2) Group DEX; and (3) Group PRO. After 1-h of anesthetic infusion, brain and lungs were removed for molecular biology analysis. Gene expression of tumor necrosis factor (TNF)-α and vascular cell adhesion molecule 1 (VCAM-1) in lung tissue (**A**,**B**) and perilesional brain area (**C**,**D**). No difference was observed between DEX and PRO groups in relation to lung tissue TNF-α and VCAM-1 gene expressions (*p* = 0.135 and *p* = 0.079 respectively). No difference was observed between DEX and PRO groups in relation to perilesional brain area TNF-α and VCAM-1 gene expressions (*p* = 0.638 and *p* = 0.999 respectively). Box plots represent the median and interquartile range of 5 animals in STROKE group and 10 animals in DEX and PRO groups. Comparisons were done by Kruskal–Wallis test followed by Dunn’s multiple comparisons test (*p* < 0.05).

Dexmedetomidine, compared to STROKE group, reduced cumulative DAD score [median (interquartile range) 12(7.8–15.3) vs. 19.5(18–24), *p* = 0.007] due to less interstitial edema [2(2–3.3) vs. 6(6–8.3), *p* = 0.017)] and hemorrhage [(2(2–3.25) vs. 6.5(4–9), *p* = 0.010]. BI was lower in dexmedetomidine than in the STROKE group [2.28(2.08–2.36) vs. 2.64(2.53–2.77)], *p* = 0.006) (Table 1) (Fig. 1). Dexmedetomidine decreased TNF-α gene expression compared to STROKE group [dexmedetomidine = 0.11(0.06–0.16) vs. STROKE = 0.95(0.76–1.22), *p* = 0.0003] (Fig. 2A). Propofol increased VCAM-1 gene expression [propofol = 2.22(2.08–3.10) vs. STROKE = 0.98(0.86–1.18), *p* = 0.0004] in lung tissue compared to STROKE group (Fig. 2B).

**Table 1:** Diffuse alveolar damage score and bronchoconstriction index.

	STROKE	DEX	PRO
**Lung parenchyma**			
Interstitial oedema (0–16)	6 (6–8.3)	2 (2–3.3)*	3 (2–4)
Haemorrhage (0–16)	6.5 (4–9)	2 (2–3.25)*	3.5 (2.75–4)
Inflammation (0–16)	2.5 (2–3.8)	2.5 (1.75–4)	4 (2–4)
Atelectasis (0–16)	4.5 (3–6)	3.5 (2–4.5)	6 (3.8–6)
Cumulative DAD (0–64)	19.5 (18–24)	12 (7.8–15.3)*	14 (11–17.8)
**Airways**			
Bronchoconstriction index	2.64 (2.53–2.77)	2.28 (2.08–2.36)*	2.57 (2.52–2.70)#

All animals were submitted to focal ischemic stroke. 24-h later, they were randomly assigned, by sealed envelopes, to (1) Group STROKE; (2) Group DEX; and (3) Group PRO. After 1-h of anesthetic infusion, lungs were removed for histology analysis. Cumulative DAD score representing injury from interstitial oedema, haemorrhage, inflammation and atelectasis. STROKE, animals submitted to stroke without dexmedetomidine or propofol infusion; DEX, animals submitted to stroke and submitted to dexmedetomidine; PRO, animals submitted to stroke and submitted to propofol. No difference was observed between PRO versus STROKE (*p* = 0.177) and PRO versus DEX (*p* = 0.404) in relation to cumulative DAD score. No difference was observed between PRO versus STROKE (*p* = 0.999) in relation to bronchoconstriction index. Values are given as medians (interquartile ranges) of 5 animals in the STROKE group and 10 animals in the DEX and PRO groups. Comparisons were done by Kruskal–Wallis test followed by Dunn’s multiple comparisons test (*P* < 0.05). vs *STROKE, #DEX.

### Neurodegeneration, ApopTag, CD45 + , CD68 + , and biological markers within perilesional brain tissue

**Figure 3:**
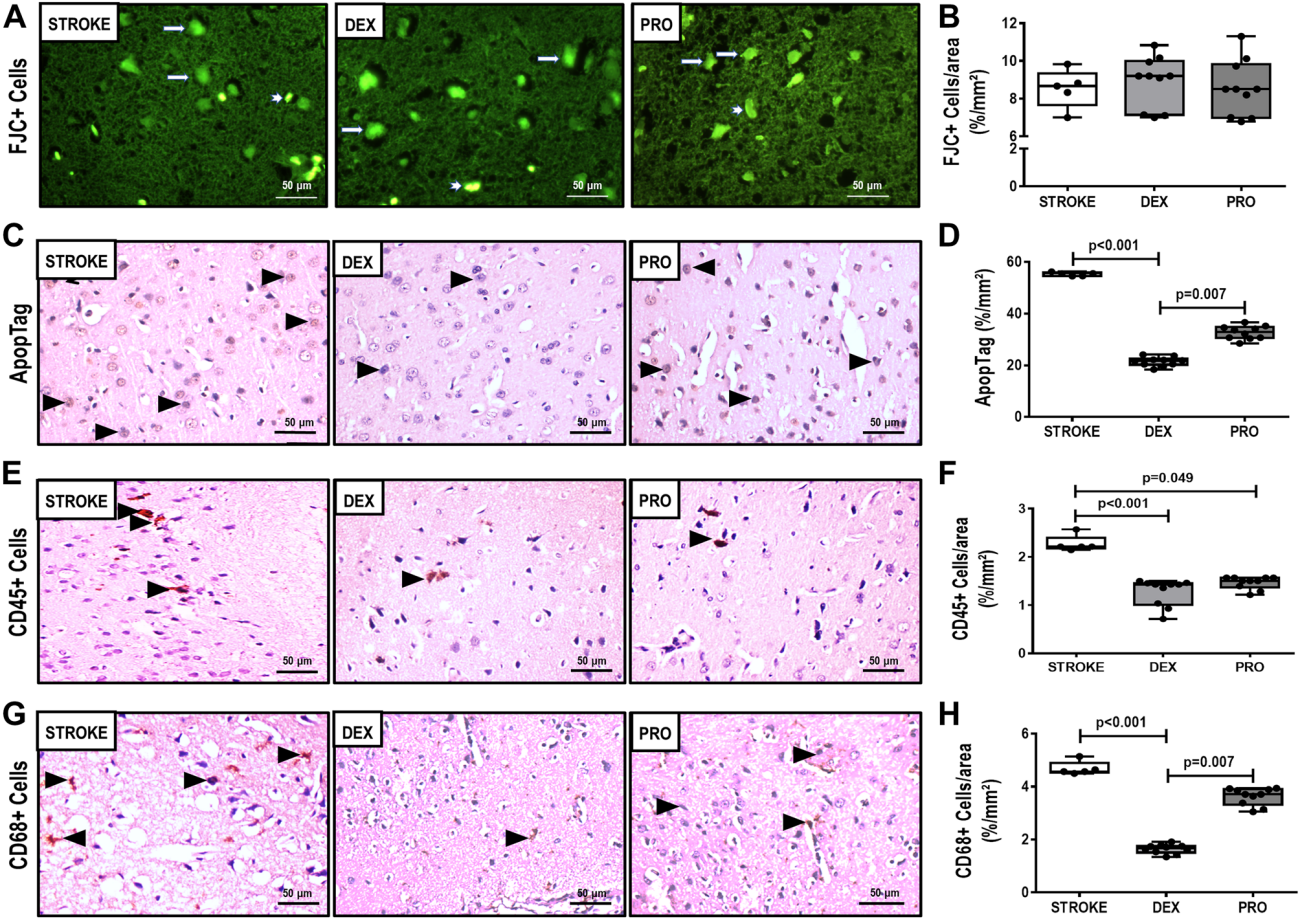
All animals were submitted to focal ischemic stroke. 24-h later, they were randomly assigned, by sealed envelopes, to (1) Group STROKE; (2) Group DEX; and (3) Group PRO. After 1-h of anesthetic infusion, brain was removed for Fluoro-Jade C staining and immunofluorescence, ApopTag kit detection, and immunohistochemical analysis for CD45 + and CD68 + cells. (**A**) Images showing staining of all degenerating neurons by FluoroJade C, which is expressed by the number of FluoroJade C-positive cells per area (%/mm^2^) in STROKE, DEX and PRO groups. (**B**) Quantification of FluoroJade C-positive cells per area (%/mm^2^). (**C**) Images showing ApopTag kit staining in STROKE, DEX, and PRO groups. (**D**) Quantification of ApopTag + cells per area (%/mm^2^). No difference was observed between STROKE and PRO groups in relation to ApopTag + cells per area (%/mm^2^) (*p* = 0.188). (**F**) Images showing CD45 + cells in STROKE, DEX and PRO groups. (**E**) Quantification of CD45 + cells per area (%/mm^2^). No difference was observed between DEX and PRO groups in relation to CD45 + cells per area (%/mm^2^) (*p* = 0.247). (**F**) Images showing CD68 + cells in STROKE, DEX and PRO groups. (**G**) Quantification of CD68 + cells per area (%/mm^2^). No difference was observed between STROKE and PRO groups in relation to ApopTag + cells per area (%/mm^2^) (*p* = 0.188). Box plots represent the median and interquartile range of 5 animals in STROKE, DEX, and PRO groups. Comparisons done by Kruskal–Wallis test followed by Dunn’s multiple comparisons test (*p* < 0.05).

Neurodegeneration did not differ among groups (Fig. 3A,B). The DEX group presented a reduction in the percentage of apoptotic cells in comparison to STROKE and PRO (*p* < 0.001, and *p* = 0.007, respectively) (Fig. 3C,D). Both DEX and PRO groups presented reduced CD45 + cells (Fig. 3E,F), whereas DEX alone showed decreased CD68 + cells compared to STROKE and PRO groups (*p* < 0.001, and *p* = 0.007, respectively) (Fig. 3G,H). Dexmedetomidine decreased TNF-α gene expression compared to STROKE group [DEX = 0.07(0.01–0.46) vs. STROKE = 0.94(0.83–1.24), *p* = 0.010] (Fig. 2C). Propofol increased VCAM-1 gene expression [propofol = 1.93(1.29–2.93) vs. STROKE = 1.00(0.78–1.30), *p* = 0.024] in perilesional brain tissue compared to STROKE group (Fig. 2D).

### Primarily isolated alveolar macrophages and lung endothelial cells after stroke

**Figure 4:**
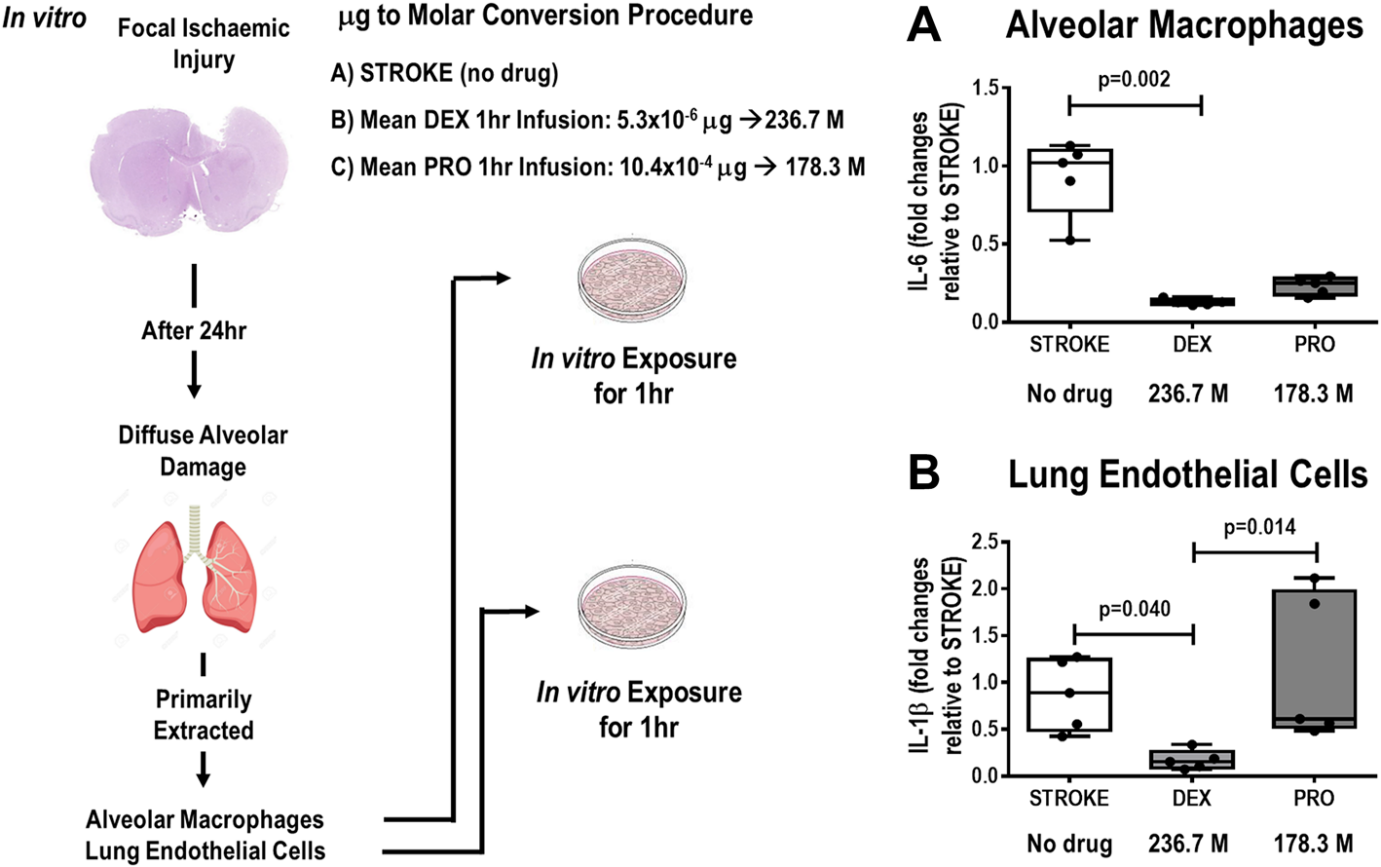
Overall in vivo and in vitro experimental scheme. Left: For the in vivo experiments, in order to obtain comparable work of breathing and mean arterial pressure, different total drug infusion concentrations of dexmedetomidine and propofol were infused (mean values from 10 animals: 5.3 × 10^–6^ μg, and 10.4 × 10^–4^ μg, respectively). Right: For the in vitro experiments, the total drug infusion concentrations of both dexmedetomidine and propofol were converted to respective plasma molarity (236.7 M and 178.3 M). An additional 5 male Wistar rats were subjected to focal ischemic stroke; 24 h later, alveolar macrophages and lung endothelial cells were primarily extracted, cultured, and, after achieving 80% confluence, exposed to similar amounts of dexmedetomidine and propofol as used in the in vivo experiments. Gene expressions of IL-6 in alveolar macrophage (**A**) and IL-1β in lung endothelial cells (**B**) in the STROKE, DEX, and PRO groups were evaluated after 1 h of exposure, the same time used for the in vivo experiments. No difference was observed between PRO versus STROKE (*p* = 0.198) and PRO versus DEX (*p* = 0.312) in relation to IL-6 in alveolar macrophages. No difference was observed between PRO versus STROKE (*p* = 0.999) in relation to IL-1β in lung endothelial cells. Box plots represent the median and interquartile range of 5 animals in each group. Comparisons done by Kruskal–Wallis test followed by Dunn’s multiple comparisons test (*p* < 0.05).

Figure 4 depicts the study design of in vitro studies based on in vivo protocol. Primarily isolated cells were exposed for 1-h to dexmedetomidine and propofol (236.7 M and 178.3 M, respectively) equivalent to mean plasma concentrations obtained in in vivo study. In alveolar macrophages and endothelial cells, IL-6 [DEX = 0.12(0.11–0.14) vs. STROKE = 1.02(0.71–1.10), *p* = 0.002] and IL-1β [DEX = 0.15(0.08–0.26) vs. STROKE = 0.88(0.48–1.24), *p* = 0.040] expressions were lower after dexmedetomidine exposure compared to STROKE. In addition, in endothelial cells, IL-1β was lower in dexmedetomidine than propofol [DEX = 0.15(0.08–0.26) vs. PRO = 0.61(0.52–1.97), *p* = 0.014] (Fig. 4A,B).

## Discussion

In the model of AIS used herein, the following was observed in lungs: (1) cumulative DAD score, BI, TNF-α gene expression, and percentage of apoptotic cells were reduced in DEX, while VCAM-1 gene expression was increased in PRO, compared to STROKE; and (2) CD45 + cells were reduced in both DEX and PRO, while CD68 + cells were decreased only in DEX. In perilesional brain tissue, TNF-α expression was decreased in DEX group and VCAM-1 expression was increased in PRO. In alveolar macrophages and lung endothelial cells, IL-6 and IL-1β expressions were decreased in DEX compared to STROKE. In lung endothelial cells, IL-1β expression was increased in PRO compared to DEX. Our data suggest that dexmedetomidine, compared to propofol, may act at the cellular level, contributing towards reduction of lung damage 24-h after AIS. Nonetheless, these results could also be influenced by the short action of dexmedetomidine on reducing TNF-α expression and percentage of apoptotic, CD45 +, and CD68 + cells within perilesional brain tissue.

To our knowledge, this is the first study to compare dexmedetomidine with propofol in rat AIS, focusing on both brain and lung gene expressions of inflammatory markers. Focal AIS was induced by thermocoagulation of pial vessels covering the somatosensory, motor, and primary sensorimotor cortices; in addition to sensorimotor dysfunction, this causes local inflammation and blood–brain barrier damage^9^. Dexmedetomidine and propofol were chosen because of previous literature suggesting CNS protection^2,3^ and systemic immunomodulatory effects^14,15^. Moreover, they are widely used in intensive care for sedation^16^. Although no differences were observed in neurodegeneration rate within penumbra among groups in the present study, TNF-α was reduced in DEX, while PRO increased VCAM-1 compared to STROKE. FJC is able to stain any cause of neuronal death^17^. There are different types of neuronal cell death, which may depend on timing of evaluation^18^. The ApopTag kit used herein reveals focal in situ staining inside early apoptotic nuclei and apoptotic bodies, which correlates directly with the more typical biochemical and morphological aspects of apoptosis. Dexmedetomidine has been associated with CNS protection through inhibition of presynaptic norepinephrine or glutamate release in the brain^2^, as well as the PI3K/AKT, ERK1/2^19^, NF-κB/COX-2 pathways^20^, and modulation of the neuroendocrine system^21^. In fact, NF-κB/COX-2 is a canonical pathway of TNF-α expression and may explain its reduced expression within perilesional brain area after dexmedetomidine exposure. The number of CD68 + cells is known to increase over 24-h after ischemic stroke^22^, and it is a common marker for reactive microglia and macrophages^23^. Microglia can increase inflammatory mediators, such as TNF-α, which can reactivate microglia in a positive feedback mechanism^24^. Here, CD68 + cells were decreased in DEX compared to STROKE and PRO (*p* < 0.001, and *p* = 0.007, respectively), which may be associated with a reduction in TNF-α expression in brain tissue. On the other hand, despite controlling intracranial pressure, expression of p75NTR, which is related with neurotoxicity effects of propofol in traumatic brain injury^7^, can increase in pathological states related to neural cell death^25^, and as a response to that, pro-proliferative signaling cascades can be elicited, during which VCAM-1 may be involved^26^. This partly may explain the increase in VCAM-1 after propofol infusion within perilesional brain area.

Brain-lung crosstalk represents a complex interaction which may lead to important complications, increasing morbidity and mortality rates^12^. In ischemic stroke, release of pro-inflammatory mediators contributes to a state of systemic and lung inflammation^9^. In the present study, dexmedetomidine, but not propofol, reduced lung damage and airway constriction. Propofol is known to cause bronchoconstriction during anesthetic induction, perhaps due to its metabisulfite preservative and stimulation of cholinergic reflex^27^. Conversely, dexmedetomidine has bronchodilator properties and may reverse histamine effects, preventing bronchospasm in experimental animals^28^. Alveolar macrophage and lung endothelial cell exposure to dexmedetomidine, compared to STROKE, reduced IL-6 and IL-1β gene expressions. These beneficial effects have only been shown in vivo^29,30^. The likely mechanism may rely on different pathways: (1) inhibition of signaling by NF-κB, a central mediator of inflammation that regulates pro-inflammatory (IL-6, IL-1β TNF-α) and antiapoptotic (Bcl2, TRAF-1, XIAP, caspase-3) pathways^20,31^; (2) inhibition of NLRP3 inflammasome activity, as previously observed in hyperoxia-induced lung injury^32^; and (3) mediation, at least in part, by α2-adrenergic receptors through the cholinergic anti-inflammatory pathway, by both vagal- or α7nAChR-dependent mechanisms^33^.

In the current experiment, animals were maintained in spontaneous ventilation to avoid confounding factors associated with ventilator-induced lung injury (e.g., barotrauma and biotrauma)^34^, as we aimed to evaluate the effects of dexmedetomidine or propofol in animals exposed to acute ischemic stroke with no further influence of pulmonary changes or hemodynamic instability. Lung mechanics, respiratory rate, and mean arterial pressure did not differ between the groups. This is consistent with the anesthetic plan, which was standardized across groups to minimize any risk of bias associated with increased inspiratory effort, hypoxemia, or ventilator asynchrony. The observation time was short (1-h); thus, we did not assess protein levels of biological markers. However, this observation time was sufficient for measurement of gene expression of key markers and early apoptosis. The 24-h period following stroke induction was chosen to evaluate drug intervention based on a previous study by our group^9^ that assessed the time course of the pulmonary inflammatory response secondary to AIS.

This study has some limitations. First, neurofunctional status of our animals were not evaluated due to the nature of our study design. However, important biomarkers associated with inflammation and endothelial cell damage within perilesional brain area as well as neurodegeneration were analyzed. Second, as a specific AIS model was used, our data cannot be extrapolated to other models or different severities of brain injury. Third, rats were healthy, young, and male, and data may not be directly extrapolated to the complexity of the clinical scenario, in which comorbidities are common. Finally, only animals with acute ischemic stroke were studied, since the immunomodulatory effects of dexmedetomidine and propofol in physiologic and models of inflammatory injury conditions have been widely investigated^2,6,15,19,33,35–37^.

## Conclusion

In the experimental model of acute ischemic stroke used herein, dexmedetomidine, but not propofol, was associated with brain and lung protection.

## Methods

### Study approval

This study was approved by the Ethics Committee with the Use of Animals (CEUA-CCS-017/17) of the Health Sciences Centre of the Federal University of Rio de Janeiro (UFRJ), Rio de Janeiro, Brazil. All animals received care in accordance with the “Principles of Laboratory Animal Care”, formulated by the National Society for Medical Research and the “*Guide for the Care and Use of Laboratory Animals”* from the National Academy of Sciences, USA. This study followed the ARRIVE guidelines for reporting of animal research^38^. Conventional animals were housed at a controlled temperature (23 °C) and controlled light–dark cycle (12–12 h), with free access to water and food, while no acclimation was done.

### Animal preparation and experimental protocol

Twenty-five healthy male Wistar rats (body weight: 340 ± 36 g) were anesthetized [xylazine 2.5 mg kg^−1^, intraperitoneally (i.p.) and ketamine 75 mg kg^−1^, i.p.], placed in a stereotactic frame with their heads immobilized, and then underwent ischemic stroke by thermocoagulation of pial blood vessels that cover the somatosensory, motor and primary sensorimotor cortex^9^. The procedure consists of craniotomy following the skin incision, exposing the left frontoparietal cortex (+ 2 to − 6 mm A.P. to bregma). Transdural thermocoagulation of blood in the superficial vessels was performed by approaching a hot probe adjusted to 300 °C temperature^39^. Vessel lesions were evaluated macroscopically through the colour changes after 5 min. The incision tissue was sutured, and animals were kept warm under a heating lamp returning to their cages after recovery from anesthesia. The stroke procedure was performed by the same experienced investigator (G.C.S.), guaranteeing the same stroke severity pattern.

After 24-h, all animals were anesthetized (sodium thiopental 50 mg kg^−1^, ip), and an intravenous (i.v.) catheter (JELCO 24G, Becton, Dickinson and Company, USA) was inserted into the tail vein, and continuous anesthesia was initiated according to randomization, done by sealed envelopes: (1) STROKE group (n = 5)—animals did not receive infusion of dexmedetomidine or propofol, but only additional sodium thiopental; (2) DEX group (n = 10)—dexmedetomidine (PRECEDEX, Laboratories Abbott do Brasil Ltda., São Paulo, SP, Brazil) was administered with a bolus of 5 μg kg^−1^ i.v. for 10 min and then an infusion of 0.1–0.5 μg kg^−1^ h^−1^ i.v. for 50 min; (3) PRO group (n = 10)—propofol (PROPOVAN, Laboratories Cristália from Brazil Ltda., Itapira, São Paulo, SP, Brazil) was administered with a bolus of 100–200 μg kg^−1^ min^−1^ i.v. for 10 min and then infusion of 100–400 μg kg^−1^ min^−1^ i.v. for 50 min (Supplemental Material, Fig. S1). Both infusion rates were based on previous studies that showed no adverse hemodynamic effects in rats during infusion for 60 min^36,37^. Anesthetics were titrated according to the evaluation of the anesthetic plan through mean arterial pressure (MAP) and clinical parameters (responses to light, movement in response to stimulus and by loss of the righting reflex: once placed in a supine position, the animal will not spontaneously revert to the prone position)^40,41^.

Upon reaching an adequate anesthetic plan, the animals were placed in the supine position, following subcutaneous anesthesia with 1% lidocaine (0.4 mL), a midline cervical incision and tracheostomy were performed. A catheter (18G; Arrow International, USA) was placed in the right internal carotid artery for arterial blood gas analysis (ABL80 FLEX; Radiometer Medical, Denmark), and MAP monitoring (Networked Multiparameter Veterinary Monitor LifeWindow 6000 V; Digicare Animal Health, Florida, USA).

The animals were kept on spontaneous breathing with supplemental oxygen (1 L/min), which provides an estimated FiO_2_ of 25%. Airflow, airway, and esophageal pressures were measured by respective pressure transducers and continuously recorded by custom-made software written in LabVIEW (National Instruments, Austin, TX). Tidal volume (V_T_) was calculated by digital integration of the flow signal. The minute pressure–time product (PTP/minute), which denotes work of breathing per minute; and the esophageal pressure generated 100 ms after onset of inspiratory effort (P_0.1_) were analyzed. The peak and mean transpulmonary pressures were gathered. All respiratory variables were analyzed by a routine written in MATLAB (Version R2007a; The Mathworks Inc, Natick, MA, USA).

Functional data were obtained immediately after venous access and animal preparation (INITIAL) and after 60 min of continuous anesthetic infusion at spontaneous ventilation (FINAL) (Supplemental Material, Fig. S1). At FINAL, heparin (1000 IU) was injected into the tail vein and animals were euthanized by overdose of sodium thiopental (150 mg/kg). The lungs and brain were carefully removed for histology and molecular biology analysis.

### Prior presentations

 The protocol and results of this study have been presented in part at the American Thoracic Society (ATS) online scientific meeting in Philadelphia in 2020 and were previously published as an abstract.

## Histology

### Lung histology

The left lung was fixed in 4% buffered formalin and embedded in paraffin. Sections (3 μm thick) were stained with hematoxylin and eosin. Photomicrographs at magnifications of × 25, × 100, and × 400 were obtained from four nonoverlapping fields of view per section using a light microscope. Diffuse alveolar damage (DAD) was quantified using a weighted scoring system to represent the severity of interstitial edema, hemorrhage, inflammatory infiltration, and atelectasis, defining 0 as for no effect and 4 for maximum severity^9,42^. The extension of each feature was graded with 0 standing for no appearance and 4 for complete involvement. Scores were calculated as the product of severity and extent of each feature, ranging from 0 to 16. The cumulative DAD score ranged from 0 to 64^9,43^.

Using a magnification of × 400, ten distal airways from each animal were viewed and the degree of bronchoconstriction index (BI) was calculated as NI/√NP^44^, in which NI stands for number of intercepts with epithelial basal membrane and NP is the number of points falling on the airway lumen. Lung histology analysis was performed by one investigator (M.V.F.) blinded to group allocation.

### Brain histology

Brain was extracted and fixed in 4% paraformaldehyde for 24 h. After serial dehydration the material was paraffin-embedded and set aside for microtome.

#### Fluoro-Jade C staining and immunofluorescence

Fluoro-Jade C (FJC) staining is widely used for the specific detection of all degenerating mature neurons, regardless of cell death mechanisms (e.g. apoptosis, necrosis)^17^. Brains were fixed in formalin (10%) for 18 h at 4 °C. They were then cut coronally into 5-mm sections, processed in paraffin, cut serially at 6 μm thickness on a rotary microtome, and mounted on positively charged slides. After drying at room temperature for 24 h, sections were dewaxed in xylene, hydrated, kept in an overnight protein bath (5% bovine albumin in phosphate-buffered saline) to minimize autofluorescence, and permeabilized with 0.5% Triton-X100 in phosphate-buffered saline for 15 min. A slightly modification was made to the protocol established by Schmued et al.^17^, and described elsewhere^45^. Briefly, sections were then immersed in 70% ethanol for 2 min, followed by a bath in 1% NaOH in 80% ethanol for 5 min, and rinsed consecutively in 70% ethanol and distilled water (2 min each). Then, sections were incubated in 0.06% potassium permanganate for 15 min, rinsed again in distilled water (1 min), and transferred to a 0.0001% solution of Fluoro-Jade C in 0.1% acetic acid, freshly prepared from aqueous stock solution (0.01%), for 15 min. Finally, sections were rinsed in distilled water, mounted with acid glycerin mounting medium, and observed in an epifluorescence microscope (Eclipse E800, Nikon, Japan).

### Apoptotic cells detection

Apoptotic cells were detected via DNA fragmentation using the terminal deoxynucleotidyl transferase (Tdt) dUTP Nick-End Labeling (TUNEL) assay (S7101, Apoptag Plus Peroxidase in situ Apoptosis Detection Kit, Merck-Millipore, MA, EUA). The DNA strand breaks were detected by enzymatically labeling the free 3′-OH termini with modified nucleotide, as instructed by the manufacturer.

### Immunohistochemistry

**Primary antibodies:** mouse anti-rat CD68 (MCA341R, ED1, Biorad AbDSerotec Ltd, UK), mouse anti rat CD45 (554875, OX-1, BD Pharmingen, CA, USA).

**Secondary antibody:** Universal immunoperoxidase polymer for rat tissue sections (N-Histofine single stain rat MAX PO (414191F, anti-mouse, anti-rabbit primary antibodies, Nichirei Biosciences Inc, Japan).

**Protocol:** After dewaxing and rehydrating paraffin sections, endogenous peroxidase was inhibited (3% H_2_O_2_ in methanol). Sections were subjected to heat-mediated antigen retrieval in a microwave oven (power 600 W) for 3 min in sodium citrate buffer (10 mM) pH 6.0 for both antibodies. After cooling, the sections were incubated with PBS containing 5% bovine serum albumin (BSA) and normal 5% goat serum (1 h) in a humid chamber at room temperature. Samples were then incubated in primary antibodies for approximately 20 h in the refrigerator. Then, the sample was incubated with peroxidase-conjugated secondary antibody (N-Histofine single stain rat MAX PO, for anti-mouse, and anti-rabbit primary antibodies; 414191F, Nichirei Biosciences Inc, Japan) for 1 h, washed with 0.25% Tween 20-phosphate saline buffer (PBS), and developed with the chromogenic substrate diaminobenzidine (Liquid DAB, Dako). After repeated washing, sections were counterstained with hematoxylin. Negative controls were performed by incubating the histological sections with nonimmune rabbit or mouse serum in place of the primary antibody. Quantification of apoptotic cells and immunohistochemistry were performed by one investigator (C.M.S.).

### Histomorphometric analysis

Semiquantitative data were derived from high-resolution photomicrographs (2048 × 1536 pixel buffer) acquired using a digital camera (Evolution VF, Media Cybernetics Inc., Bethesda, MD, USA) connected to a Nikon Eclipse E-800 light microscope and Q-capture 2.95.0 software (Silicon Graphic Inc., Milpitas, CA, USA). Ten non-overlapping images of perilesional brain areas were taken. Quantification of Fluoro-Jade C positive cells was performed by one investigator (C.T.) and monitored by a second investigator (G.S.S.) blinded to group assignment. Data are shown as degenerated cells per perilesional brain area (cells/mm^2^)^41^.

### In vitro experiments

Additional 5 healthy Wistar male rats (body weight: 345 ± 30 g) underwent acute ischemic stroke and, after 24 h, alveolar macrophages and lung endothelial cells were primarily isolated using immunomagnetic bead-based method using specific antibodies (CD11b and CD54, respectively). Alveolar macrophages were obtained from bronchoalveolar lavage fluid (BALF), and endothelial cells were obtained from lung tissue single cells isolate. BALF was collected after 3 mL of PBS was flushed into the lungs (3 times of 1 ml). BALF was centrifuged at 250 × g at 4 °C for 10 min. The cell pellet contains BALF cells. Lungs were then cut into small pieces, incubated with collagenase I 1% (Sigma-Aldrich) for 40 min at 37 °C under agitation for cell dissociation. After digestion, cells were passed through a 100 μm cell strainer and washed with PBS 1X. According to manufacturer’s protocol, BALF cells and lung single cells isolate were incubated with biotinylated antibodies anti-CD11b (BIOLEGEND) or anti-CD54 (BIOLEGEND) for 20 min on ice, washed, then incubated with Dynabeads biotin binder (ThermoFisher Scientific) for 20 min on ice. Cells were washed, and isolated after exposure to magnetic fields. Cells were plated in 10^5^ cells /well in a 6 well plate (coated with gelatin 0.2%, when endothelial cells) in humidified adequate CO_2_ incubator (5% CO_2_, 95% N_2_). After achieving 80% of confluence, cells were exposed for 1-h to equivalent mean plasma concentrations (Molar) of DEX or PRO used in in vivo experiments for the 10 animals or no drug, which served as control. Gene expressions of markers associated to inflammation (IL-6, IL-1β) were evaluated in alveolar macrophages and lung endothelial cells, respectively, by quantitative real-time reverse transcription polymerase chain reaction (RT-PCR).

### Biological markers in brain and lung tissues

RT-PCR was performed to measure biological markers associated with inflammation [tumor necrosis factor (TNF)-α] and endothelial cell damage [vascular cell adhesion molecule (VCAM-1)] in perilesional brain tissue and central slices of right lung. Inflammatory markers associated to inflammation [interleukin (IL)-6, and IL-1β] were evaluated on primarily isolated alveolar macrophages and lung endothelial cells.

Lung total RNA was extracted from frozen tissues with RELIAPREP RNA Tissue Miniprep System (Promega Corporation, Fitchburg, Wisconsin, USA), following manufacturer recommendations. RNA concentration was measured by spectrophotometry in a NANODROP ND-2000 system (Thermo Fisher Scientific, Wilmington, DE). First-strand cDNA was synthesized from total RNA using the High-capacity cDNA Reverse Transcription Kit (Thermofisher, Massachusetts, USA). Relative mRNA levels were measured with a BRYT Green system (Promega, Fitchburg, WI) using PCR Mastercycler ep Realplex Eppendorf (Eppendorf, Hamburg, Germany). Samples were measured in triplicate.

Perilesional area RNA was isolated from the paraffin-embedded brain tissue with the MAGMAX FFPE DNA/RNA Ultra Kit (ThermoFisher Scientific, Massachusetts, USA**).** RNA was recovered, cDNA was obtained by the already described methods and quantitative RT-PCR was performed to measure biomarkers. The primers are shown in Supplemental Material, Table S4.

For each sample the expression of each gene was normalized to the acidic ribosomal phosphoprotein P0 (*36B4*)^46^ housekeeping gene and expressed as the fold change relative to STROKE, using the 2^−ΔΔCt^ method, where ΔCt = Ct (target gene) − Ct (reference gene)^47^. Blinded analyses were carried out by one investigator (M.A.A.).

### Statistical analysis

The sample size was calculated to allow detection of differences between dexmedetomidine and STROKE in TNF-α levels of brain injured rats^48^. The number of 10 animals per group was calculated based on a power of 80%, α = 5%, two-tailed, and effect size (*d*) of 1.37 (G*Power 3.1.9.2; University of Düsseldorf, Düsseldorf, Germany). The primary outcome was the gene expression of TNF-α in lung tissue, whereas the secondary outcomes were lung function, DAD score, BI, neurodegeneration, expression of markers related to inflammation and endothelial cell damage.

The ventilatory pattern and arterial blood gas data (INITIAL and FINAL) were analyzed by the Two-way ANOVA test followed by the Holm-Šidák multiple comparisons tests to compare parameters among groups and overtime. For analysis of parametric and non-parametric data obtained at the end of the experiment, one-way ANOVA followed by Holm-Šidák multiple comparisons test or Kruskal–Wallis test followed by Dunn’s multiple comparisons test were performed (*p* < 0.05). Parametric data was expressed as mean ± standard deviation (SD) and non-parametric data, as median (interquartile range). All tests were performed in GraphPad Prism version 8.00 (GraphPad Software, La Jolla, CA, USA). Significance was established at *p* < 0.05.

## Supplementary Information

Supplementary Information.
