# Supplementary material for: Comparative effects of dexmedetomidine and propofol on brain and lung damage in experimental acute ischemic stroke
Source: Sci Rep. 2021 Nov 30;11:23133. doi: 10.1038/s41598-021-02608-1 (PMC8633001; doi:10.1038/s41598-021-02608-1)
Supplement: Supplementary file 1 — Supplementary Information. [file 41598_2021_2608_MOESM1_ESM.docx]

**Supplement**

**Comparative effects of dexmedetomidine and propofol**

**on brain and lung damage in experimental acute ischemic stroke**

Giselle C Sousa^a,b^, MD; Marcos Vinicius Fernandes^a,c^, MS; Fernanda F Cruz^a,c^, MD, PhD; Mariana A Antunes^a^, PhD; Carla M da Silva^a,d^, MSc; Christina Takyia^d^, MD, PhD; Denise Battaglini^e^, MD; Cynthia S Samary^a,c^, PhD; Chiara Robba^e,f^, MD, PhD; Paolo Pelosi^e,f^, MD, FERS; Patricia RM Rocco^a,c^*, MD, PhD; Pedro L Silva^a,c^* PhD

*PRMR and PLS shared senior authorship.

^a^Laboratory of Pulmonary Investigation, Institute of Biophysics Carlos Chagas Filho, Federal University of Rio de Janeiro, Rio de Janeiro, RJ, Brazil.

^b^Department of Anesthesiology, Federal University of Rio de Janeiro, Rio de Janeiro, Brazil.

^c^Rio de Janeiro Network on Neuroinflammation, Carlos Chagas Filho Foundation for Supporting Research in the State of Rio de Janeiro (FAPERJ)

^d^Laboratory of Imunopathology, Institute of Biophysics Carlos Chagas Filho, Federal University of Rio de Janeiro, Rio de Janeiro, RJ, Brazil.

^e^San Martino Policlinico Hospital, IRCCS for Oncology and Neurosciences, University of Genoa, Genoa, Italy

^f^Department of Surgical Sciences and Integrated Diagnostics (DISC), University of Genoa, Genoa, Italy

*Correspondence and reprint requests to*: Prof. Pedro Leme Silva, Ph.D. Laboratory of Pulmonary Investigation, Carlos Chagas Filho Biophysics Institute, Federal University of Rio de Janeiro, Centro de Ciências da Saúde, Avenida Carlos Chagas Filho, 373, Bloco G-014, Ilha do Fundão, Rio de Janeiro, RJ 21941-902, Brazil. Tel: +55 21 3938-6530 / Fax: +55 21 2280-8193. Email: pedroleme@biof.ufrj.br

**Table S1: Respiratory variables and hemodynamic at INITIAL and FINAL**

|  | Groups | INITIAL | FINAL | Time Effect | Group Effect | Interaction |
| --- | --- | --- | --- | --- | --- | --- |
| **V_T_ (mL/kg)** |  |  |  | p=0.966 | p=0.788 | p=0.717 |
|  | **DEX** | 4.9 ± 1.0 | 4.8 ± 1.0 |  |  |  |
|  | **PRO** | 4.9 ± 0.9 | 5.0 ± 0.8 |  |  |  |
| **RR (bpm)** |  |  |  | p=0.221 | p=0.514 | p=0.019 |
|  | **DEX** | 76 ± 11 | 87 ± 13 |  |  |  |
|  | **PRO** | 81 ± 7 | 77 ± 12 |  |  |  |
| **Flow (mL/s)** |  |  |  | p=0.140 | p=0.484 | p=0.301 |
|  | **DEX** | 8.9 ± 1.7 | 9.1 ± 1.6 |  |  |  |
|  | **PRO** | 9.2 ± 2.0 | 10.1 ± 2.7 |  |  |  |
| **V′_E_ (mL/min)** |  |  |  | p=0.311 | p=0.798 | p=0.164 |
|  | **DEX** | 123 ± 22 | 141 ± 29 |  |  |  |
|  | **PRO** | 136 ± 27 | 133 ± 28 |  |  |  |
| **Ppeak,_L_ (cmH_2_O)** |  |  |  | p=0.007 | p=0.756 | p=0.639 |
|  | **DEX** | 6.9 ± 1.7 | 8.8 ± 2.5 |  |  |  |
|  | **PRO** | 7.4 ± 2 | 8.8 ± 2.8 |  |  |  |
| **Pmean,_L_ (cmH_2_O)** |  |  |  | p=0.046 | p=0.233 | p=0.195 |
|  | **DEX** | 2.3 ± 1.0 | 3.0 ± 1.2 |  |  |  |
|  | **PRO** | 3.1 ± 0.9 | 3.2 ± 0.9 |  |  |  |
| **P_0.1_ (cmH_2_O)** |  |  |  | p=0.083 | p=0.319 | p=0.217 |
|  | **DEX** | -1.5 ± 1.4 | -2.5 ± 1.9 |  |  |  |
|  | **PRO** | -2.5 ± 1.0 | -2.7± 1.4 |  |  |  |
| **PTP minute (cmH_2_O.seg/min)** |  |  |  | p=0.678 | p=0.465 | p=0.603 |
|  | **DEX** | 122 ± 89 | 137 ± 55 |  |  |  |
|  | **PRO** | 116 ± 30 | 114 ± 29 |  |  |  |
| **MAP (mmHg)** |  |  |  | p=0.028 | p=0.128 | p=0.833 |
|  | **DEX** | 112 ± 26 | 101 ± 23 |  |  |  |
|  | **PRO** | 126 ± 17 | 113 ± 17 |  |  |  |
| **Total dose of anesthetic (μg)** |  |  |  |  |  |  |
|  | **DEX** | - | 5.3±2.2x10^-6^ |  |  |  |
|  | **PRO** | - | 10.4± 3.9x10^-4^ |  |  |  |

**Table S1:** Respiratory variables and hemodynamics obtained in INITIAL and FINAL, V_T_: tidal volume; RR: respiratory rate; V′_E_: minute volume; Ppeak,_L_: peak transpulmonary pressure; Pmean,_L_: mean transpulmonary pressure; P_0.1_: esophageal pressure 100 ms after the beginning of the respiratory effort; PTP minute: pressure product time per minute; MAP: mean arterial pressure. Variables represent the mean ± standard deviation (SD) of 10 animals per group. Comparisons were done by Two-way ANOVA test followed by the Holm-Šídák multiple comparisons test (p<0.05).

**Table S2: Arterial blood gases**

|  | Groups | INITIAL | FINAL | Time Effect | Group Effect | Interaction |
| --- | --- | --- | --- | --- | --- | --- |
| **pHa** |  |  |  | p=0.135 | p=0.766 | p=0.019 |
|  | **DEX** | 7.44 ± 0.05 | 7.45 ± 0.05 |  |  |  |
|  | **PRO** | 7.46 ± 0.04 | 7.41 ± 0.07 |  |  |  |
| **PaO_2_ (mmHg)** |  |  |  | p=0.454 | p=0.360 | p=0.797 |
|  | **DEX** | 79 ± 34 | 90 ± 38 |  |  |  |
|  | **PRO** | 101 ± 68 | 107 ± 64 |  |  |  |
| **PaCO_2_ (mmHg)** |  |  |  | p=0.879 | p=0.885 | p=0.023 |
|  | **DEX** | 41 ± 9 | 36 ± 8 |  |  |  |
|  | **PRO** | 36 ± 6 | 40 ± 7 |  |  |  |
| **HCO_3_ (mmol/L)** |  |  |  | p=0.832 | p=0.816 | p=0.531 |
|  | **DEX** | 27± 4 | 27 ± 7 |  |  |  |
|  | **PRO** | 26 ± 3 | 27 ± 6 |  |  |  |
| **BE (mmol/L)** |  |  |  | p=0.214 | p=0.468 | p=0.992 |
|  | **DEX** | 2.8 ± 2,7 | 1.5 ± 3.8 |  |  |  |
|  | **PRO** | 2.2 ± 2,6 | 0.9 ± 1.9 |  |  |  |

**Table S2:** Arterial blood gases analysis obtained at INITIAL and FINAL. PaO_2_: arterial oxygen partial pressure; PaCO_2_: arterial carbon dioxide partial pressure; HCO_3_: Bicarbonate; BE: base excess. Variables represent mean ± standard deviation (SD) of 10 animals per group. Comparisons were done using Two-way ANOVA test followed by the Holm-Šídák multiple comparisons test (p<0.05).

**Table S3. Diffuse alveolar damage score and bronchoconstriction index.**

|  | SHAM | STROKE |
| --- | --- | --- |
| Lung parenchyma |  |  |
| Interstitial oedema (0–16) | 1 (0.3–1.8) | 6 (6–8.3)† |
| Haemorrhage (0–16) | 0.8 (0–1.8) | 6.5 (4–9)† |
| Inflammation (0–16) | 0.5 (0–1.8) | 2.5 (2–3.8) |
| Atelectasis (0–16) | 2 (2–3.5) | 4.5 (3–6) |
| Cumulative DAD (0–64) | 4.5 (4.0–5.3) | 19.5 (18–24)† |
| Airways |  |  |
| Bronchoconstriction index | 1.90 (1.80–2.02) | 2.64 (2.53–2.77)† |

**Table S3:** Cumulative DAD score representing injury from interstitial oedema, haemorrhage, inflammation and atelectasis. SHAM, animals submitted to surgical procedure without stroke as well as dexmedetomidine or propofol infusion; STROKE, animals submitted to stroke without dexmedetomidine or propofol infusion. Values are given as medians (interquartile ranges) of 5 animals in the SHAM and STROKE groups. Comparisons were done by Mann-Whitney test (*P*<0.05). *vs* †SHAM.

**Table S4: Primers for quantitative real-time reverse transcription polymerase chain reaction**

**Forward and reverse oligonucleotide sequences of target gene primers**

| **Gene** | **Primer** | **Primer sequences (5′-3′)** |
| --- | --- | --- |
| ***Lung*** | | |
| TNF-α | Forward | TTG CTT CTT CCC TGT TCC |
|  | Reverse | CTG GGC AGC GTT TAT TCT |
| VCAM-1 | Forward | TGC ACG GTC CCT AAT GTG TA |
|  | Reverse | TGC CAA TTT CCT CCC TTA AA |
| ***Brain*** | | |
| TNF-α | Forward | TTG CTT CTT CCC TGT TCC |
|  | Reverse | CTG GGC AGC GTT TAT TCT |
| VCAM-1 | Forward | TGC ACG GTC CCT AAT GTG TA |
|  | Reverse | TGC CAA TTT CCT CCC TTA AA |
| ***Endothelium*** | | |
| IL-1β | Forward | CTA TGT CTT GCC CGT GGA G |
|  | Reverse | CAT CAT CCC ACG AGT CAC A |
| ***Macrophage*** | | |
| IL-6 | Forward | CTC CGC AAG AGA CTT CCA G |
|  | Reverse | CTC CTC TCC GGA CTT GTG A |
| ***House Keeping*** | | |
| 36B4 | Forward | AAT CCT GAG CGA TGT GCA G |
|  | Reverse | GCT GCC ATT GTC AAA CAC C |

IL1-β: interleukin 1 beta; TNF-α: tumor necrosis factor alpha; VCAM-1: vascular cell adhesion molecule-1; IL-6: interleukin 6; 36B4, acidic ribosomal phosphoprotein P0.

**Figure S1: A.** Experimental design. All animals were submitted to focal ischemic stroke. 24-hours later, they were randomly assigned, by sealed envelopes, to 1) Group CTRL (n = 5) – animals did not receive dexmedetomidine or propofol; 2) Group DEX (n = 10) – anesthetized with dexmedetomidine (Precedex®, Laboratories Abbott do Brazil Ltda., São Paulo, SP, Brazil) with a bolus of 5 μg.kg^-1^ for 10 min and then an infusion of 0.1 -0.5 μg.kg^-1^.h^-1^ for 50 min; 3) Group PRO (n = 10) - anesthetized with propofol (Propovan®, Laboratories Cristália do Brasil Ltda., Itapira, São Paulo, SP, Brazil), initial infusion of 100-200 μg.kg^-1^.min^-1^ for 10 min and then infusion of 100-400 μg.kg^-1^.min^-1^ for 50 min. **Figure S1:** **B.** Timeline of the experiments. I.p.: Intraperitoneal; s.c.: subcutaneous; ABG: arterial blood gas.


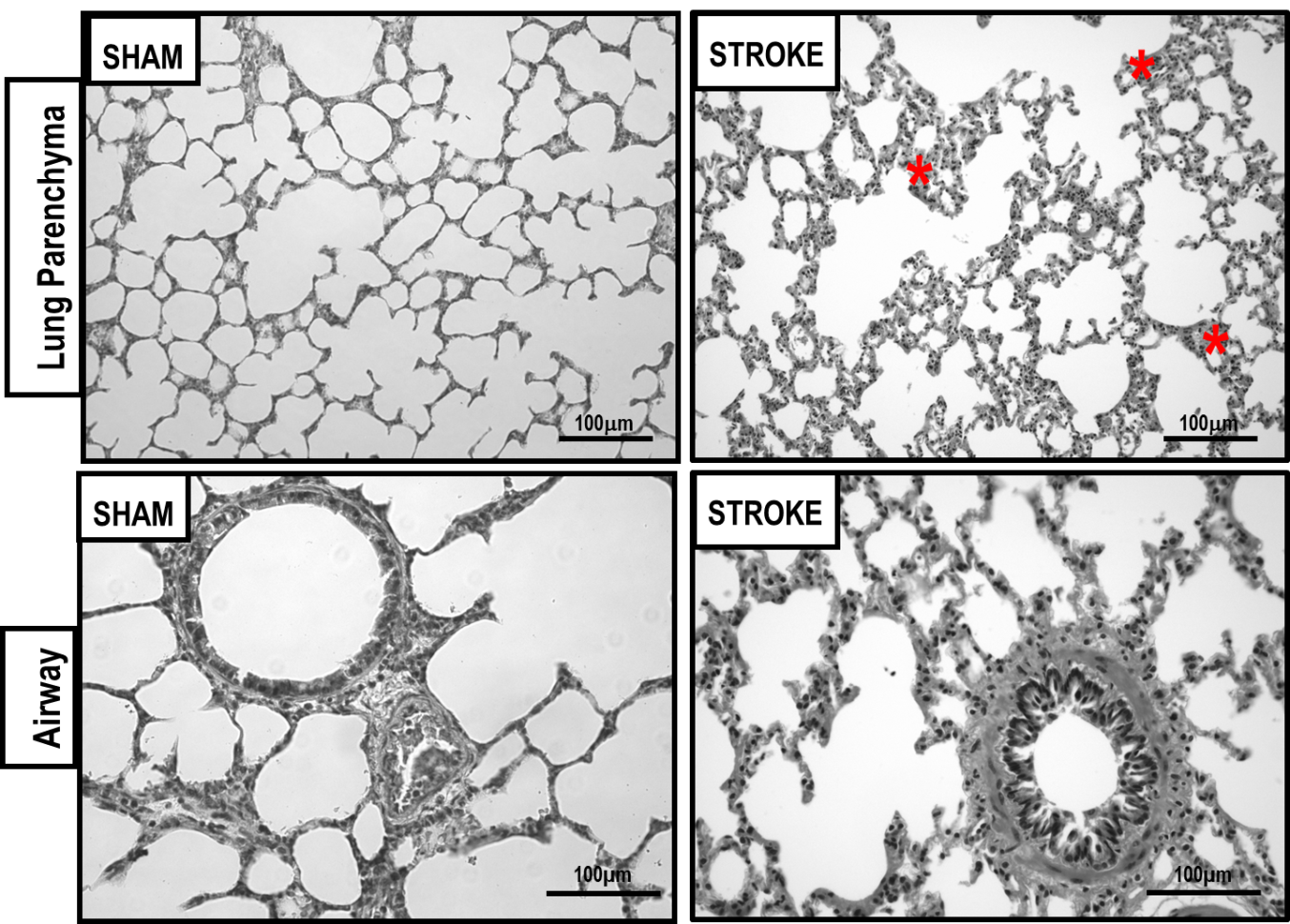


**Figure S2:** Representative images of lung parenchyma upon autopsy in SHAM (animals submitted to surgical procedure without stroke as well as dexmedetomidine or propofol infusion) and STROKE group (only stroke without dexmedetomidine or propofol infusion), and airway (inferior panels, x400). Note the atelectasis (red asterisks) in the STROKE. The airway lumen in STROKE was constricted compared to SHAM group. Scale bar = 100μm


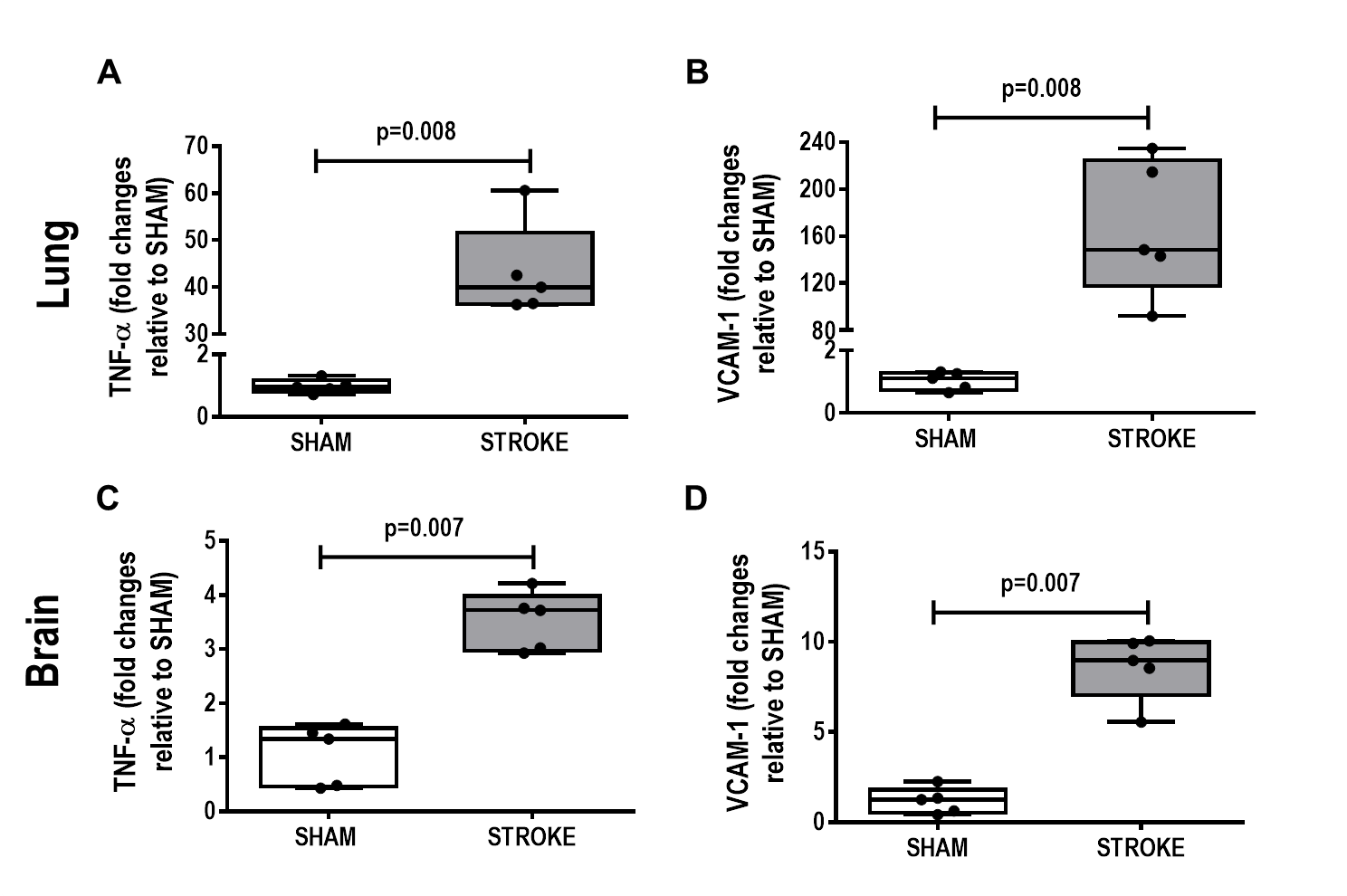


**Figure S3:** Gene expression of tumor necrosis factor (TNF)-α and vascular cell adhesion molecule 1 (VCAM-1) in lung tissue (A, and B) and perilesional brain area (C, and D) in SHAM and STROKE groups. Box plots represent the median and interquartile range of 5 animals in SHAM and STROKE groups. Comparisons were done by Mann-Whitney test (p<0.05).


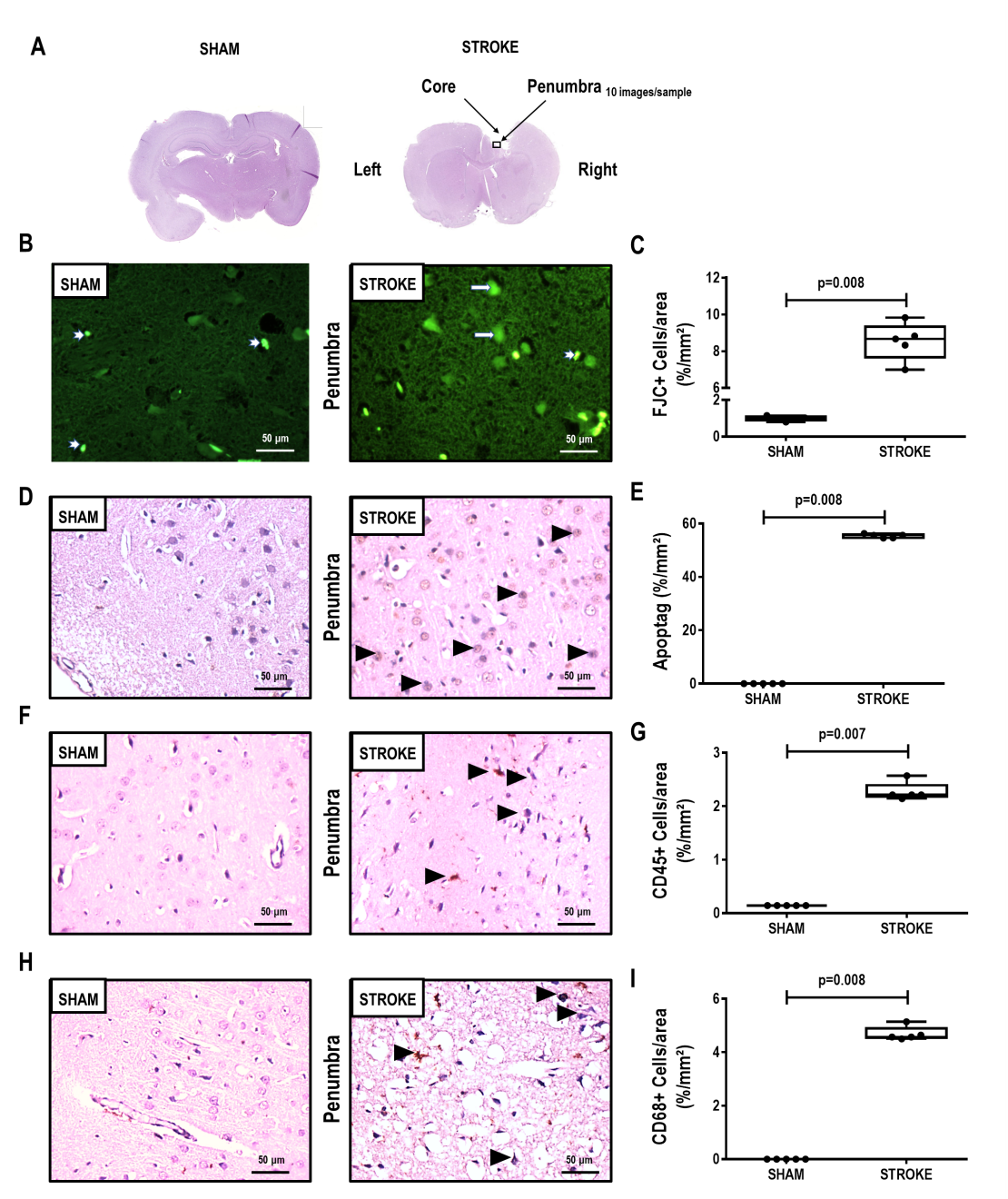


**Figure S4**: A: Scanned brains from SHAM and STROKE groups. Scheme showing the core and the brain perilesional area (Penumbra), where 10 non-overlapping images were gathered. B: Images showing stain all degenerating neurons by FluoroJade C in SHAM and STROKE groups. C: Quantification of FluoroJade C+ cells per area (%/mm^2^). D: Images showing ApopTag kit stain in SHAM and STROKE groups. E: Quantification of ApopTag+ cells per area (%/mm^2^). F: Images showing CD45+ cells in SHAM and STROKE groups. G: Quantification of CD45+ cells per area (%/mm^2^). H: Images showing CD68+ cells in SHAM and STROKE groups. I: Quantification of CD68+ cells per area (%/mm^2^). Box plots represent the median and interquartile range of 5 animals in SHAM and STROKE groups. Comparisons were done by Mann-Whitney test (p<0.05).
